# Imageless navigation for primary total hip arthroplasty: a meta-analysis study

**DOI:** 10.1186/s10195-022-00636-9

**Published:** 2022-04-15

**Authors:** Filippo Migliorini, Francesco Cuozzo, Francesco Oliva, Joerg Eschweiler, Frank Hildebrand, Nicola Maffulli

**Affiliations:** 1 Department of Orthopaedic, Trauma, and Reconstructive Surgery, RWTH University Hospital, 52074 Aachen, Germany; 2 Department of Medicine, Surgery and Dentistry, University of Salerno, 84081 Baronissi, SA Italy; 3 School of Pharmacy and Bioengineering, Keele University Faculty of Medicine, Stoke on Trent, ST4 7QB UK; 4 Queen Mary University of London, Barts and the London School of Medicine and Dentistry, Centre for Sports and Exercise Medicine, Mile End Hospital, London, E1 4DG UK

**Keywords:** Hip, Arthroplasty, Navigated, Imageless navigation

## Abstract

**Background:**

There has been a growing interest in imageless navigation for primary total hip arthroplasty (THA). Its superiority over standard THA is debated. This meta-analysis compared surgical duration, implant positioning, Harris Hip Score and rate of dislocation of imageless navigation versus conventional THA.

**Methods:**

The present study was conducted according to the PRISMA 2020 guidelines. All the clinical trials comparing imageless navigation versus conventional for primary THA were accessed. In January 2022, the following databases were accessed: PubMed, Web of Science, Google Scholar and Embase. No time constraints were used for the search. The outcomes of interest were to compare cup inclination and anteversion, leg length discrepancy, surgical duration, Harris Hip Score and rate of dislocation of imageless navigation versus conventional THA.

**Results:**

Twenty-one studies (2706 procedures) were retrieved. Fifty-two percent of patients were women. There was between-group comparability at baseline in terms of age, body mass index (BMI), visual analogue scale, Harris Hip Score and leg length discrepancy (*P* > 0.1). Compared with conventional THA, the navigated group demonstrated slightly lower leg length discrepancy (*P* = 0.02) but longer duration of the surgical procedure (*P* < 0.0001). Cup anteversion (*P* = 0.6) and inclination (*P* = 0.5), Harris Hip Score (*P* = 0.1) and rate of dislocation (*P* = 0.98) were similar between the two interventions.

**Conclusion:**

Imageless navigation may represent a viable option for THA.

## Background

Total hip arthroplasty (THA) is considered one of the most effective and successful interventions in orthopaedics [1–3]. Alignment of the acetabular component and limb length equalization are essential for a successful THA. Cup inclination of 45° ± 10° and cup anteversion of 15° ± 10° are considered the safe zone by most surgeons [4, 5]. Malposition of the acetabular component increases the risk of dislocation, impingement, pelvic osteolysis, acetabular migration and acetabular lining wear [6–9]. Greater leg length discrepancy is associated with back pain, gait impairment, higher rate of aseptic loosening and patient dissatisfaction [9–14].

Imageless navigation system, a computer-assisted procedure, exploits three-dimensional sensors positioned during surgery to detect the anatomical features, limb axes and joint orientation, avoiding exposure to X-rays [15]. Imageless navigated THA is believed to be more accurate than other techniques, as the use of these sensors is believed to overcome parallax or operator pitfalls with fluoroscopy or change in pelvis position during the operation [16, 17]. However, whether imageless navigated THA is superior to the conventional procedure in terms of implant positioning and clinical and functional outcomes remains controversial [18–22]. Several studies have been recently published and had not yet been considered in previous meta-analyses. Therefore, a meta-analysis was conducted to compare imageless navigation versus conventional for primary THA in terms of implant positioning, surgical duration, leg length difference, Harris Hip Score and rate of dislocation.

## Material and methods

### Eligibility criteria

All the clinical trials comparing imageless navigation versus conventional THA were accessed. Only studies with levels I–III of evidence, according to the Oxford Centre of Evidence-Based Medicine [23], were eligible. Only studies published in peer-reviewed journals were considered. According to the authors’ language capabilities, articles in English, German, Italian, French and Spanish were eligible. Animal, in vitro, biomechanics, computational and cadaveric studies were not eligible. Reviews, opinions, letters and editorials were not considered. Clinical studies which reported data on revision THA were not included. Studies combining the interventions with innovative implants or materials, or experimental rehabilitation programmes, were not considered. Only studies which reported the outcomes of imageless navigation were suitable; other types of navigation methods [e.g. robotic, computed tomography (CT)-based] were not eligible. Only studies which reported quantitative data under the outcomes of interest were eligible.

### Search strategy

This systematic review followed the Preferred Reporting Items for Systematic Reviews and Meta-Analyses (PRISMA) 2020 checklist [24]. The PICO algorithm was followed:

- P (Population): end-stage hip osteoarthrosis;
- I (Intervention): conventional THA;
- C (Comparison): imageless navigated THA;
- O (Outcomes): implant positioning, surgical duration, Harris Hip Score, dislocations.

In January 2022, the following databases were accessed: PubMed, Web of Science, Google Scholar and Embase. No time constraints were set for the search. The following keywords were used for the database search: hip, osteoarthritis, pain, replacement, arthroplasty, prosthesis, lower limb, leg discrepancy, anteversion, inclination, radiological, complications, dislocations, Harris Hip Score, PROMs, patient reported outcome measures.

### Selection and data collection

Two authors (F.M. and F.C.) independently performed the database search. Titles and abstract of interest were screened. The full text of the articles of interest was accessed. The bibliography of the full-text articles was also screened for inclusion. Disagreements were debated, and the final decision was taken by N.M.

### Data items

Data extraction was performed by two authors independently (F.M. and F.C.) (Table 1). The following generalities and patient demographics were collected: author and year, journal, study design, number of procedures, number of women, mean age and BMI, type of intervention and surgical approach. The following data were retrieved at last follow-up: mean cup inclination and anteversion, surgical duration, leg length discrepancy, Harris Hip Score [25] and rate of dislocation.

**Table 1:** Typology of data extracted at baseline, baseline and last follow-up (FU), and last FU

Data at baseline	Data at baseline and at last FU	Data at last FU
Name of first author Year of publication Journal of publication Included patients (*n*) Age (mean, years) Body mass index (mean, kg/m^*2*^) Women (%) Length of follow-up (months) Range of motion (mean, degrees)	Leg length discrepancy (cm) Harris Hip Score (0–100)	Cup inclination (mean, degrees) Cup anteversion (mean, degrees) Surgical duration (mean, min) Rate of dislocation (number of events)

### Methodological quality assessment

The risk of bias assessment was performed by two authors (F.M. and F.C.) independently. The Cochrane risk of bias tool (The Nordic Cochrane Collaboration, Copenhagen) was used. The following biases were evaluated: selection, detection, attrition, reporting, other source of biases.

### Synthesis methods

The statistical analyses were performed by the main author (F.M.). For descriptive statistics, IBM SPSS version 25 was used. The Shapiro–Wilk test was performed to investigate data distribution. For parametric data, mean and standard deviation were evaluated. For non-parametric data, median and interquartile ranges were evaluated. Mean difference (MD) effect measure was adopted to assess baseline comparability. Student’s *t*-test and Mann–Whitney *U* test were performed for parametric and non-parametric data, with *P* values > 0.1 considered satisfactory. For the meta-analyses, Review Manager 5.3 software (The Nordic Cochrane Collaboration, Copenhagen) was used. For continuous data, the inverse variance with MD effect measure was adopted, while the Mantel–Haenszel method with odds ratio (OR) effect measure was used for dichotomic data. Heterogeneity was investigated using the Higgins *I*^2^ and *χ*^2^ tests. If *χ*^2^ < 0.05 and *I*^2^ > 75%, high heterogeneity was found. A fixed method effect model was used as default; if high heterogeneity was found, a random effect model was used. The confidence interval (CI) was set at 95% in all comparisons. Overall *P* values of < 0.05 were considered statistically significant.

## Results

### Study selection

**Fig. 1:**
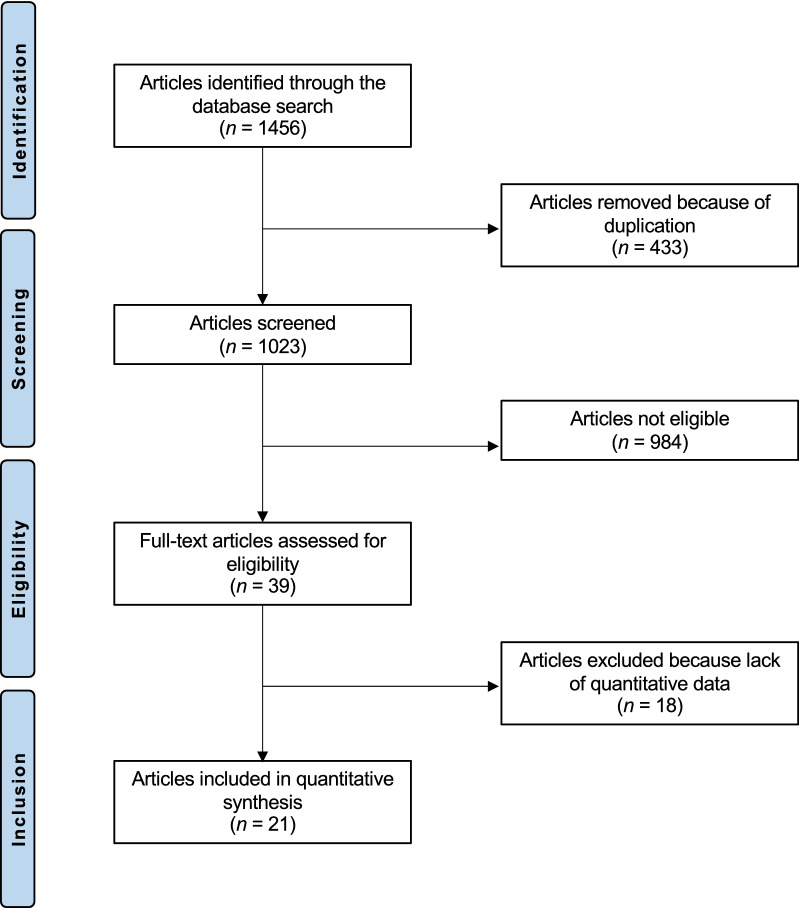
Flow chart of the literature search

The literature search resulted in 1456 articles. After removal of duplicates (*N* = 433), a further 984 articles were not eligible: non-comparative clinical trial (*N* = 571), study design (*N* = 219), experimental material/protocols (*N* = 84), other type of navigation system (*N* = 77), short length of follow-up (*N* = 18), not peer reviewed (*N* = 11) or language limitations (*N* = 4). Additionally 18 studies were excluded as they missed quantitative data under the endpoints of interest. Finally, 21 studies were included: 10 randomized clinical trials (RCTs) and 3 prospective and 8 retrospective cohort trials (Fig. 1). The time interval covered by the studies was from 2005 to 2018.

### Methodological quality assessment

**Fig. 2:**
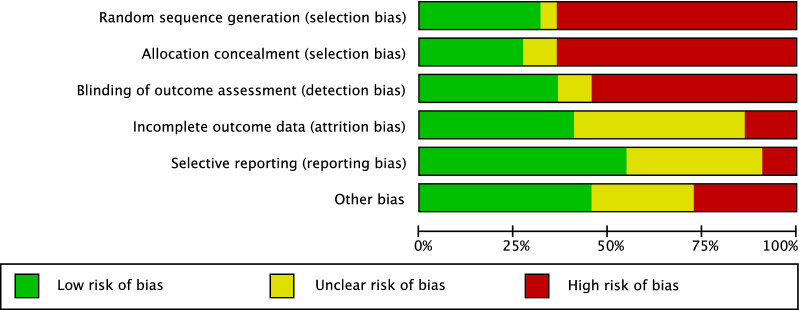
Methodological quality assessment

The risk of bias graph evidenced moderate risk of selection bias, which arose from the retrospective nature of 8/21 of included studies (Fig. 2). Given the lack of blinding methods by most of the included studies, the risk of detection bias was moderate. Attrition and reporting biases were also moderate, as was the risk of other biases.

### Risk of publication bias

**Fig. 3:**
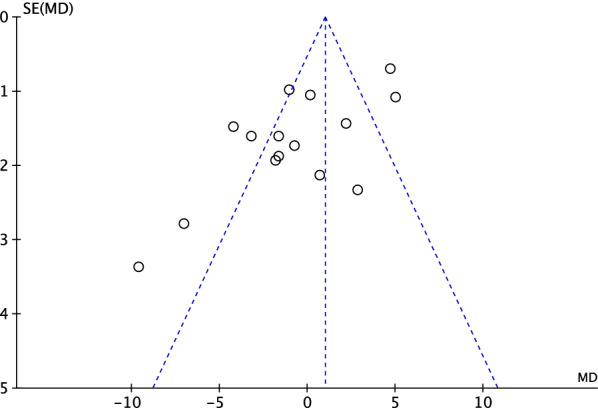
Funnel plot of the most reported outcome (cup anteversion)

To evaluate the risk of publication bias, the funnel plot of the most commonly reported outcome (cup anteversion) was evaluated. The plot evidenced fair symmetrical disposition of the referral points, which may indicate moderate risk of publication bias. However, most of the referral points are contained within the range of acceptability (Fig. 3).

### Study characteristics and results of individual studies

Overall, 2706 procedures were retrieved, of which 52% were in women. The mean age was 64.0 ± 4.2 years. The mean BMI was 27.7 ± 1.8 kg/m^2^. There was between-group comparability at baseline in terms of age, BMI, Harris Hip Score and leg length discrepancy (*P* > 0.1). Generalities and patient baseline of the included studies are shown in greater detail in Table 2.

**Table 2:** Generalities and patient baseline of the included studies; RCT, randomized clinical trial

Author, year	Journal	Design	Procedures (*n*)	Female gender	Mean age	Mean BMI	Intervention
Brown et al., 2014 [26]	*J Arthroplasty*	Retrospective	119	57%	63.6	30.0	Imageless navigated
			198	54%	64.4	30.0	Conventional
Ellapparadja et al., 2016 [19]	*Hip Int*	Retrospective	152	54%	67.0	29.0	Imageless navigated
			57	79%	72.0	28.8	Conventional
Gurgel et al., 2014 [27]	*J Arthroplasty*	RCT	20	50%	51.3	27.4	Imageless navigated
			20	15%	54.0	27.5	Conventional
Hohmann et al., 2011 [28]	*J Arthroplasty*	RCT	30		66.5	30.0	Imageless navigated
			91		67.8	30.7	Conventional
Kalteis et al., 2005 [29]	*Int Orthop*	RCT	23	65%	63.5	28.0	Imageless navigated
			22	59%	62.4	28.7	Conventional
Kalteis et al., 2006 [30]	*Bone Joint J*	RCT	30	60%	63.1	27.6	Imageless navigated
			30	57%	64.7	28.5	Conventional
Lass et al., 2014 [31]	*J Arthroplasty*	RCT	62	66%	65.6	27.6	Imageless navigated
			63	54%	68.9	27.0	Conventional
Licini et al., 2013 [17]	*Orthopedics*	Retrospective	75		67.0		Imageless navigated
			75		66.1		Conventional
Lin et al., 2011	*Orthopedics*	Prospective	25	48%	62.1	26.5	Imageless navigated
			25	40%	63.5	28.8	Conventional
Mainard et al., 2008 [32]	*Orthopedics*	RCT	42	57%	63.3		Imageless navigated
			42	48%	60.5		Conventional
Manzotti et al., 2009 [33]	*Int Orthop*	Retrospective	48		72.2		Imageless navigated
			48		72.0		Conventional
Nam et al., 2013 [34]	*Orthopedics*	Retrospective	90	45%	58.7	29.3	Imageless navigated
			90	59%	67.5	28.7	Conventional
			90	60%	68.0	28.4	Conventional
Najarian et al., 2009 [5]	*J Arthroplasty*	Retrospective	55		65.0	27.0	Conventional
			50		64.0	28.0	Imageless navigated
			50		65.0	29.0	Imageless navigated
Oh et al., 2018 [20]	*J Orthop Surg*	Retrospective	30	15%	62.2	23.1	Imageless navigated
			30	15%	62.1	24.2	Conventional
Ottersbach et al., 2005 [35]	Z Orthop Grenzgeb	Prospective	50	66%	59.2		Imageless navigated
			50	56%	60.3		Conventional
Parratte et al., 2007 [36]	*Rev Chir Orthop*	RCT	30	53%	61.2	25.6	Imageless navigated
			30	53%	62.6	25.2	Conventional
Parratte et al., 2016 [22]	*Clin Orthop Rel Res*	RCT	30	53%	61.2	25.6	Imageless navigated
			30	53%	62.6	25.2	Conventional
Renkawitz et al., 2016 [21]	*Bone Joint J*	RCT	66	56%	62.5	26.9	Imageless navigated
			69	50%	62.9	27.1	Conventional
Sendtner et al., 2010 [37]	*Int Orthop*	RCT	32	59%	68.0	28.0	Imageless navigated
			30	63%	70.0	26.0	Conventional
Shah et al., 2017 [18]	*J Arthroplasty*	Prospective	202	54%	67.2	29.5	Imageless navigated
			173	46%	58.3	30.2	Conventional
Wixson et al., 2005 [38]	*J Arthroplasty*	Retrospective	82		64.0		Imageless navigated
			50		61.0		Conventional

### Results of syntheses

**Fig. 4:**
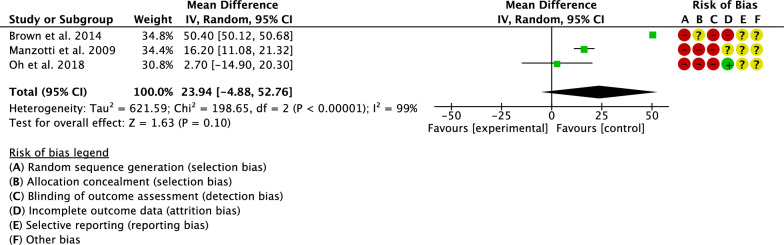
Meta-analysis of the comparison: surgical duration

Three studies compared the surgical duration [20, 26, 33]. The final effect was not significant (*P* = 0.1), indicating no difference between the two groups (Fig. 4).

**Fig. 5:**
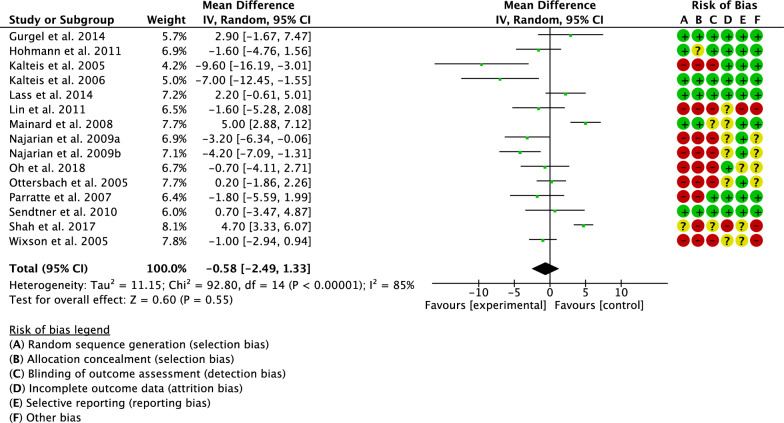
Meta-analysis of the comparison: cup anteversion

Cup anteversion has been evaluated by 15 studies [5, 18, 20, 27–32, 35–38]. The final effect was not significant (*P* = 0.6), indicating no difference between the two groups (Fig. 5).

**Fig. 6:**
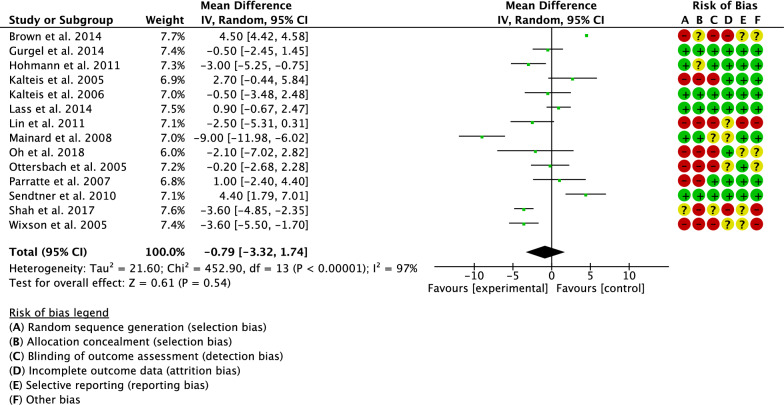
Meta-analysis of the comparison: cup inclination

Cup inclination has been evaluated by 14 studies [18, 20, 26–32, 35–38]. The final effect was not significant (*P* = 0.5), indicating no difference between the two groups (Fig. 6).

**Fig. 7:**
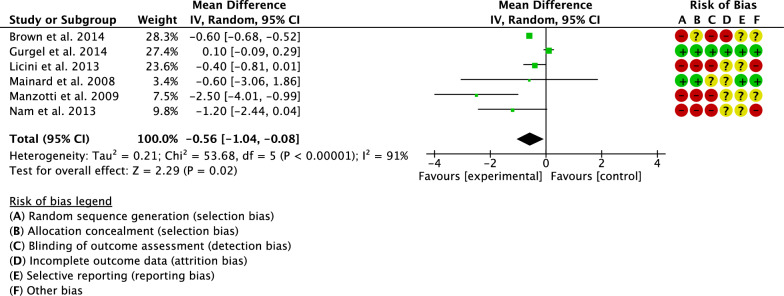
Meta-analysis of the comparison: leg length discrepancy

Six studies compared leg length discrepancy [17, 26, 27, 32–34]. Leg length discrepancy was lower in the navigated group (MD −0.56; 95% CI −1.04 to −0.08; *P* = 0.02; Fig. 7).

**Fig. 8:**
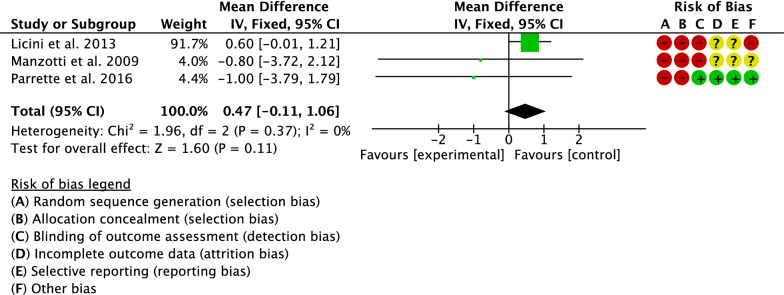
Meta-analysis of the comparison: Harris Hip Score

Three studies compared the Harris Hip Score [17, 22, 33]. The final effect was not significant (*P* = 0.1), indicating no difference between the two groups (Fig. 8).

**Fig. 9:**
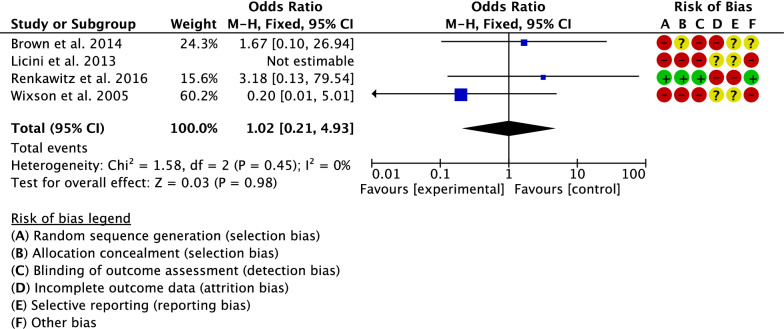
Meta-analysis of the comparison: dislocation

The rate of dislocation has been compared by four studies [17, 21, 26, 38]. The final effect was not significant (*P* = 0.98), indicating no difference between the two groups (Fig. 9).

## Discussion

According to the endpoints evaluated in the present study, imageless navigation was not superior to the standard technique for primary THA. Although leg length discrepancy was significantly lower in imageless navigated THA, given its minimal difference, the clinical relevance of this finding is limited. Cup positioning, surgical duration, Harris Hip Score and rate of dislocation were similar between the two procedures. Snijders et al. [39] compared 255 versus 259 patients who underwent navigated versus freehand THA, respectively. The authors reported that navigated THA is more precise and exhibits an improved cup positioning accuracy [39] compared with the freehand group. Similarly, a meta-analysis on the accuracy of cup positioning revealed that imageless THA is preferable to traditional methods [40]. The present study found no evidence of greater accuracy implant in positioning in favour of the navigated THA. The present study expanded the focus also to other endpoints (implant positioning, surgical duration, Harris Hip Score, dislocations), but found no difference between the two modalities. These findings call into question the benefit of imageless THA, especially in terms of cost.

Although all the studies reported a longer surgical duration in the imageless navigated THA, the comparison was weighted by the high level of heterogeneity, and no statistically difference was found between the two groups. Imageless navigated THA is more time demanding, especially during the pre-operative phases [26]. The first incision is performed to place the optical tracking arrays in the iliac crest [26]. Additional time is required to position, drape and prep the patient again. Further, pelvic and acetabulum registration are the last steps which precede the beginning of the surgery [26]. In contrast, in conventional THA, the skin incision identified the start of the operation time [26]. This meta-analysis found similar cup anteversion between the two procedures. Several authors reported similar cup anteversion between the two THA groups [22, 27, 28]. Gurgel et al. [27] reported a greater anteversion of 17° in the THA navigated group compared with 15° of the conventional group. In contrast, Kalteis et al. [29] found 14° of anteversion in the imageless navigated group and 24° in the conventional THA group. Similar considerations can be made for cup inclination. Two authors reported approximately 3° of difference between the THA groups [28, 29]. Other authors reported similar cup positioning, with less than 0.5° of difference in inclination [30, 35, 37]. In contrast, Mainard et al. [32] reported a cup inclination of 44° in the imageless navigated cohort compared with 53° in the conventional THA. Leg length discrepancy was similar between the two techniques. However, given the high heterogeneity and the narrow spread of the result, the clinical relevance of this finding is uncertain. Leg length difference represents the absolute value of the difference between lines drawn through the inferior aspects of both lesser trochanters and the trans-ischial tuberosity line in the anteroposterior radiography of the lower pelvis [26, 41–43]. Licini et al. [17] reported an average leg length difference of 0.3 cm in the imageless navigated cohort versus 1.8 cm for the conventional THA, showing that leg length discrepancy was lower in the THA navigated group. However, they found no difference in patients’ perception of limb-length equality [17]. Similar results were evidenced by Manzotti et al. [33]. Although the mean pre-operative leg length discrepancy was similar between the two groups, the conventional THA showed a difference of +2.6 cm on early post-operative evaluation [33]. In contrast, other authors found no relevant difference between the two cohorts [26, 27, 34]. Three studies investigated the Harris Hip Score [17, 22, 33]. All studies agreed that the two techniques provide a similar Harris Hip Score at last follow-up [17, 22, 33]. Similar considerations can be made regarding the rate of dislocation, with no difference between imageless navigated and conventional THA [17, 26, 38].

The present study has some limitations. The limited number of clinical studies and relative limited number of procedures included for analysis represent the most important limitation. Further, the retrospective design of 38% (8 of 21) [5, 17, 19, 20, 26, 33, 34, 38] of the included studies is another important limitation. Given the limited available data in the literature, the analyses were conducted irrespective of the surgical access and the type of implant. The description of the surgical technique and post-operative rehabilitation was adequate in most of studies, whereas the sample size was often too small, and the eligibility criteria were barely reported and often biased in many of the included studies. Few studies reported data on the hip function and quality of life on long-term follow-up, and few reported the mean blood loss and the need for transfusion, which we suggest are other variables to investigate. Future high-quality studies are required to define the advantages of imageless navigation for THA. Given the lack of quantitative data, we were unable to include the rate of aseptic loosening, infection, impingement or component wear in our analysis. Optimal patient demographics at baseline is crucial to obtain reliable results. Patient selection is fundamental to conduct such studies and to minimize the risk of selection bias. Uncontrolled acute disease, such as infections or neoplasms, may have great influence on the outcome and generate heterogeneous results. The analyses were conducted irrespective of the surgical access (anterior, anterolateral, lateral, posterolateral, posterior) [44, 45]. This may generate inconsistencies [8, 14, 46]. Muscular damage or detachment, capsular repair and capsulotomy may have an influence on the surgical outcome [47–49]; however, given the lack of available data, these points were not possible to analyse separately. Given the lack of available data, surgical exposure (minimally or standard invasive), type of implant, cementation and post-operative protocol were not considered for analysis [9, 50–52]. Future investigations should overcome these limitations. For a better understanding of the potential of navigation surgery in imageless THA, future studies should investigate further radiological parameters, such as the femoral and acetabular offset as well as stem alignment. High-quality studies with longer follow-up would also be beneficial to investigate range of motion, reason for revision surgery and factors influencing the learning curve.

## Conclusion

Imageless navigation may represent a viable option for primary THA. Compared with conventional THA, the navigated THA group demonstrated slightly lower leg length discrepancy but longer duration of the surgical intervention. Cup positioning, Harris Hip Score and dislocation rates were similar between the two interventions.

## Data Availability

The datasets generated during and/or analysed during the current study are available throughout the manuscript.

## Abbreviations

THA: Total hip arthroplasty
MD: Mean difference
OR: Odds ratio
CI: Confidence interval
RCTs: Randomized clinical trials

## References

[1] Reininga IH, Zijlstra W, Wagenmakers R, Boerboom AL, Huijbers BP, Groothoff JW, Bulstra SK, Stevens M (2010). Minimally invasive and computer-navigated total hip arthroplasty: a qualitative and systematic review of the literature. BMC Musculoskelet Disord.

[2] Wang Z, Hou JZ, Wu CH, Zhou YJ, Gu XM, Wang HH, Feng W, Cheng YX, Sheng X, Bao HW (2018). A systematic review and meta-analysis of direct anterior approach versus posterior approach in total hip arthroplasty. J Orthop Surg Res.

[3] Eschweiler J, Migliorini F, Siebers H, Tingart M, Rath B (2019). Biomechanical modeling and the relevance for total hip arthroplasty. Orthopade.

[4] Banaszkiewicz PA (2014) Dislocations after total hip-replacement arthroplasties. In: Classic papers in orthopaedics. pp 113–115. doi:10.1007/978-1-4471-5451-8_27

[5] Najarian BC, Kilgore JE, Markel DC (2009). Evaluation of component positioning in primary total hip arthroplasty using an imageless navigation device compared with traditional methods. J Arthroplasty.

[6] <williamson1978.pdf>.

[7] Kennedy JG, Rogers WB, Soffe KE, Sullivan RJ, Griffen DG, Sheehan LJ (1998). Effect of acetabular component orientation on recurrent dislocation, pelvic osteolysis, polyethylene wear, and component migration. J Arthroplasty.

[8] Migliorini F, Eschweiler J, Trivellas A, Rath B, Driessen A, Tingart M, Arentini P (2020). Implant positioning among the surgical approaches for total hip arthroplasty: a Bayesian network meta-analysis. Arch Orthop Trauma Surg.

[9] Migliorini F, Biagini M, Rath B, Meisen N, Tingart M, Eschweiler J (2019). Total hip arthroplasty: minimally invasive surgery or not? Meta-analysis of clinical trials. Int Orthop.

[10] <friberg1983.pdf>.

[11] Harrison MH (2005). Robert Jones, Gathorne Girdlestone and excision arthroplasty of the hip. J Bone Joint Surg Br.

[12] Tanaka R, Shigematsu M, Motooka T, Mawatari M, Hotokebuchi T (2010). Factors influencing the improvement of gait ability after total hip arthroplasty. J Arthroplasty.

[13] <amstutz1982.pdf>.

[14] Migliorini F, Trivellas A, Eschweiler J, El Mansy Y, Mazzanti MC, Tingart M, Aretini P (2020). Hospitalization length, surgical duration, and blood lost among the approaches for total hip arthroplasty: a Bayesian network meta-analysis. Musculoskelet Surg.

[15] Jia J, Zhao Q, Lu P, Fan G, Chen H, Liu C, Liu J, Chen S, Jin Z (2019). Clinical efficacy of OrthoPilot navigation system versus conventional manual of total hip arthroplasty: a systematic review and meta-analysis. Medicine.

[16] Ogawa H, Kurosaka K, Sato A, Hirasawa N, Matsubara M, Tsukada S (2020). Does an augmented reality-based portable navigation system improve the accuracy of acetabular component orientation during THA? A randomized controlled trial. Clin Orthop Relat Res.

[17] Licini DJ, Burnikel DJ, Meneghini RM, Ochsner JL (2013). Comparison of limb-length discrepancy after THA: with and without computer navigation. Orthopedics.

[18] Shah SM, Deep K, Siramanakul C, Mahajan V, Picard F, Allen DJ (2017). Computer navigation helps reduce the incidence of noise after ceramic-on-ceramic total hip arthroplasty. J Arthroplasty.

[19] Ellapparadja P, Mahajan V, Atiya S, Sankar B, Deep K (2016). Leg length discrepancy in computer navigated total hip arthroplasty—how accurate are we?. Hip Int.

[20] Oh KJ, Kim BK, Jo MI, Ahn BM (2018). Which one is more affected by navigation-assisted cup positioning in total hip arthroplasty: anteversion or inclination? A retrospective matched-pair cohort study in Asian physique. J Orthop Surg.

[21] Renkawitz T, Weber M, Springorum HR, Sendtner E, Woerner M, Ulm K, Weber T, Grifka J (2015). Impingement-free range of movement, acetabular component cover and early clinical results comparing 'femur-first' navigation and 'conventional' minimally invasive total hip arthroplasty: a randomised controlled trial. Bone Joint J..

[22] Parratte S, Ollivier M, Lunebourg A, Flecher X, Argenson JN (2016). No benefit after THA performed with computer-assisted cup placement: 10-year results of a randomized controlled study. Clin Orthop Relat Res.

[23] Howick J CI, Glasziou P, Greenhalgh T, Carl Heneghan, Liberati A, Moschetti I, Phillips B, Thornton H, Goddard O, Hodgkinson M (2011) The 2011 Oxford CEBM Levels of Evidence. Oxford Centre for Evidence-Based Medicine. https://www.cebmnet/indexaspx?o=5653

[24] Page MJ, McKenzie JE, Bossuyt PM, Boutron I, Hoffmann TC, Mulrow CD, Shamseer L, Tetzlaff JM, Akl EA, Brennan SE, Chou R, Glanville J, Grimshaw JM, Hrobjartsson A, Lalu MM, Li T, Loder EW, Mayo-Wilson E, McDonald S, McGuinness LA, Stewart LA, Thomas J, Tricco AC, Welch VA, Whiting P, Moher D (2021). The PRISMA 2020 statement: an updated guideline for reporting systematic reviews. BMJ.

[25] Singh JA, Schleck C, Harmsen S, Lewallen D (2016). Clinically important improvement thresholds for Harris Hip Score and its ability to predict revision risk after primary total hip arthroplasty. BMC Musculoskelet Disord.

[26] Brown ML, Reed JD, Drinkwater CJ (2014). Imageless computer-assisted versus conventional total hip arthroplasty: one surgeon's initial experience. J Arthroplasty.

[27] Gurgel HM, Croci AT, Cabrita HA, Vicente JR, Leonhardt MC, Rodrigues JC (2014). Acetabular component positioning in total hip arthroplasty with and without a computer-assisted system: a prospective, randomized and controlled study. J Arthroplasty.

[28] Hohmann E, Bryant A, Tetsworth K (2011). A comparison between imageless navigated and manual freehand technique acetabular cup placement in total hip arthroplasty. J Arthroplasty.

[29] Kalteis T, Handel M, Herold T, Perlick L, Baethis H, Grifka J (2005). Greater accuracy in positioning of the acetabular cup by using an image-free navigation system. Int Orthop.

[30] Kalteis T, Handel M, Bathis H, Perlick L, Tingart M, Grifka J (2006). Imageless navigation for insertion of the acetabular component in total hip arthroplasty: is it as accurate as CT-based navigation?. J Bone Joint Surg Br.

[31] Lass R, Kubista B, Olischar B, Frantal S, Windhager R, Giurea A (2014). Total hip arthroplasty using imageless computer-assisted hip navigation: a prospective randomized study. J Arthroplasty.

[32] Mainard D (2008) Navigated and nonnavigated total hip arthroplasty: results of two consecutive series using a cementless straight hip stem. Orthopedics 31 (10 Suppl 1) 19298040

[33] Manzotti A, Cerveri P, De Momi E, Pullen C, Confalonieri N (2011). Does computer-assisted surgery benefit leg length restoration in total hip replacement? Navigation versus conventional freehand. Int Orthop.

[34] Nam D, Sculco PK, Abdel MP, Alexiades MM, Figgie MP, Mayman DJ (2013). Leg-length inequalities following THA based on surgical technique. Orthopedics.

[35] Ottersbach A, Haaker R (2005). Optimization of cup positioning in THA—comparison between conventional mechanical instrumentation and computer-assisted implanted cups by using the OrthoPilot navigation system. Z Orthop Grenzgeb.

[36] Parratte S, Argenson JN, Flecher X, Aubaniac JM (2007). Computer-assisted surgery for acetabular cup positioning in total hip arthroplasty: comparative prospective randomized study. Rev Chir Orthop Reparatrice Appar Mot.

[37] Sendtner E, Schuster T, Worner M, Kalteis T, Grifka J, Renkawitz T (2011). Accuracy of acetabular cup placement in computer-assisted, minimally-invasive THR in a lateral decubitus position. Int Orthop.

[38] Wixson RL, MacDonald MA (2005). Total hip arthroplasty through a minimal posterior approach using imageless computer-assisted hip navigation. J Arthroplasty.

[39] Snijders T, van Gaalen SM, de Gast A (2017). Precision and accuracy of imageless navigation versus freehand implantation of total hip arthroplasty: a systematic review and meta-analysis. Int J Med Robot.

[40] Liu Z, Gao Y, Cai L (2015). Imageless navigation versus traditional method in total hip arthroplasty: a meta-analysis. Int J Surg.

[41] Clark CR, Huddleston HD, Schoch EP, Thomas BJ (2006). Leg-length discrepancy after total hip arthroplasty. J Am Acad Orthop Surg.

[42] Maloney WJ, Keeney JA (2004). Leg length discrepancy after total hip arthroplasty. J Arthroplasty.

[43] Wylde V, Whitehouse SL, Taylor AH, Pattison GT, Bannister GC, Blom AW (2009). Prevalence and functional impact of patient-perceived leg length discrepancy after hip replacement. Int Orthop.

[44] Chen W, Sun JN, Zhang Y, Zhang Y, Chen XY, Feng S (2020). Correction to: direct anterior versus posterolateral approaches for clinical outcomes after total hip arthroplasty: a systematic review and meta-analysis. J Orthop Surg Res.

[45] Xie J, Zhang H, Wang L, Yao X, Pan Z, Jiang Q (2017). Comparison of supercapsular percutaneously assisted approach total hip versus conventional posterior approach for total hip arthroplasty: a prospective, randomized controlled trial. J Orthop Surg Res.

[46] Migliorini F, Trivellas A, Eschweiler J, Driessen A, Lessi F, Tingart M, Aretini P (2020). Nerve palsy, dislocation and revision rate among the approaches for total hip arthroplasty: a Bayesian network meta-analysis. Musculoskelet Surg.

[47] Miranda L, Quaranta M, Oliva F, Giuliano A, Maffulli N (2021). Capsular repair vs capsulectomy in total hip arthroplasty. Br Med Bull.

[48] Abrams GD, Hart MA, Takami K, Bayne CO, Kelly BT, Espinoza Orias AA, Nho SJ (2015). Biomechanical evaluation of capsulotomy, capsulectomy, and capsular repair on hip rotation. Arthroscopy.

[49] Ometti M, Brambilla L, Gatti R, Tettamanti A, La Cava T, Pironti P, Fraschini G, Salini V (2019). Capsulectomy vs capsulotomy in total hip arthroplasty. Clinical outcomes and proprioception evaluation: study protocol for a randomized, controlled, double blinded trial. J Orthop.

[50] Migliorini F, Driessen A, Colarossi G, El Mansy Y, Gatz M, Tingart M, Eschweiler J (2020). Short stems for total hip replacement among middle-aged patients. Int Orthop.

[51] Luger M, Feldler S, Klasan A, Gotterbarm T, Schopper C (2021). The morphology of the proximal femur in cementless short-stem total hip arthroplasty: no negative effect on offset reconstruction, leg length difference and implant positioning. J Orthop Surg Res.

[52] Liu Z, Liu B, Yang H, Zhao L (2021). Staples versus sutures for skin closure in hip arthroplasty: a meta-analysis and systematic review. J Orthop Surg Res.

